# Third Delivery in a Chinese Patient With Anomalous Origin of Left Coronary Artery From Pulmonary Artery (ALCAPA): A Case Report

**DOI:** 10.1155/crog/4830284

**Published:** 2026-04-09

**Authors:** He Zhao, Dawei Zhang, Yanna Li, Zhen Fang, Nan Zhang, Jun Zhang

**Affiliations:** ^1^ Department of Obstetrics and Gynecology of Beijing Anzhen Hospital affiliated to Capital Medical University, Beijing, China; ^2^ Obstetrics and Gynecology Medical Center of Severe Cardiovascular of Beijing Anzhen Hospital affiliated to Capital Medical University, Beijing, China; ^3^ Department of Radiology of Beijing Anzhen Hospital affiliated to Capital Medical University, Beijing, China

## Abstract

**Background:**

Anomalous origin of the left coronary artery from the pulmonary artery (ALCAPA) is a rare congenital defect that poses a significant risk of myocardial ischemia and sudden death, especially during the hemodynamic stresses of pregnancy. Although surgical correction is standard, the management of asymptomatic adult‐type ALCAPA diagnosed during pregnancy remains a high‐stakes clinical challenge.

**Case Presentation:**

We report the case of a 36‐year‐old multiparous woman with incidentally diagnosed adult‐type ALCAPA during her third pregnancy. Serial echocardiography revealed moderate mitral regurgitation. The pregnancy was complicated by superimposed preeclampsia at 33 weeks. A dedicated multidisciplinary team implemented a tailored plan for labor induction at 37 weeks in a hybrid operating room, employing epidural analgesia and continuous hemodynamic monitoring. The patient underwent a successful vaginal delivery of a healthy infant. Intriguingly, mitral regurgitation transiently worsened to severe on postpartum Day 3 but significantly improved to mild by Day 7, correlating with resolution of the pregnancy‐induced volume load. Coronary CTA postpartum confirmed the diagnosis.

**Discussion:**

Multidisciplinary planning enables safe vaginal delivery in select ALCAPA pregnancies. Dynamic peripartum changes in mitral regurgitation underline the role of volume shifts and the need for serial echocardiography to guide management and time definitive surgery.

## 1. Introduction

Anomalous origin of the left coronary artery from the pulmonary artery (ALCAPA), also known as Bland‐White‐Garland syndrome, is a rare congenital cardiovascular defect that occurs in approximately 1/300,000 live births, accounting for 0.25%–0.5% of congenital heart diseases [1] ALCAPA mostly originates from the left posterior sinus at the root of the pulmonary artery, which can lead to adverse events such as hypoxemia, coronary artery steal syndrome, and myocardial ischemia. There are two types of ALCAPA syndrome [2]: the infant type and the adult type, each with different manifestations and outcomes. Infants with ALCAPA experience myocardial infarction and congestive heart failure, and approximately 90% of them die within the 1st year of life. In some cases, patients can survive past infancy and into adulthood and do not present with symptoms until later in life. Adults with ALCAPA have well‐developed collateral circulation. Surgery is the primary treatment modality so far [3]. In this paper, we report a rare case of asymptomatic ALCAPA in a woman undergoing a third pregnancy.

### 1.1. Case Report

A 36‐year‐old pregnant woman underwent prenatal echocardiography at 25 weeks of pregnancy, and she was transferred from a community hospital to our hospital due to ALCAPA. She had no symptoms before this pregnancy and had a history of vaginal delivery eight and 10 years ago, respectively, with smooth delivery processes. The weights of the newborns were 3300 and 3100 g, respectively. She regularly exercised and jogged 12 km without feeling unwell.

**Figure 1:**
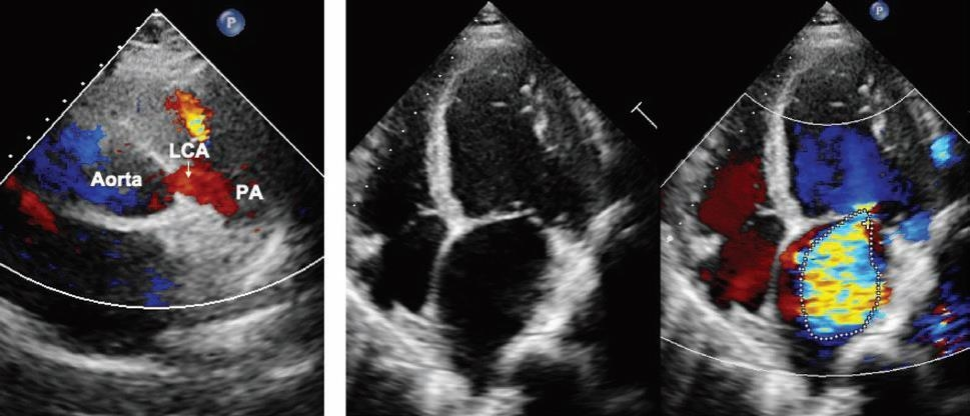
Echocardiogram showed ALCAPA (left) and mitral regurgitation (right). Left: The left coronary artery (LCA) originates from the pulmonary artery (PA). Right: The left atrium was markedly enlarged and severe mitral valve regurgitation was shown.

At the time of the visit, her blood pressure was 122/68 mmHg, pulse was 80 bpm, and heart rate was 80 bpm. ECG showed left anterior branch conduction block, and echocardiogram showed ALCAPA and mitral regurgitation (Figure 1). The BNP level was 121 pg/mL. Outpatient multidisciplinary team (MDT) management and monthly cardiac assessment were performed. Considering that she was pregnant and thus not suitable for coronary CTA or coronary angiography, a postpartum examination was scheduled. At 33 weeks of pregnancy, she was diagnosed with preeclampsia due to 24‐h ambulatory blood pressure elevation and positive urine protein, and thus treated with magnesium sulfate for spasmolysis. Blood pressure was normal and antihypertensive drugs were not required.

She was admitted to the hospital at 36 weeks of pregnancy to evaluate the method and time of delivery. Episodic supraventricular extrasystole and episodic ventricular extrasystole were found using a Holter monitor. Three MDT consultations were given. A detailed delivery plan was formulated, and it was finally decided to perform induced labor by oxytocin at 37 weeks of pregnancy. Specifically, the intravenous induction of labor is performed in the delivery room, with ECG monitoring throughout the entire process. Once in labor, the anesthesiologist performs labor analgesia and quickly transfers her to the hybrid operating room, actively assisting in the second stage of labor. In case of myocardial infarction, sudden death, and so on, emergency coronary angiography and open‐heart surgery should be performed immediately. Finally, she returns to the postpartum care unit.

The patient received induced labor by oxytocin at 6:00 on the day of the 37th week of pregnancy. At 8:05, the membrane spontaneously ruptured. At 12:20, a tube was placed outside the dura mater. At 14:00, epidural analgesia was initiated (infusion solution: a mixture of 0.1% ropivacaine and 0.4 *μ*g/mL sufentanil), and 80 mg of phloroglucinol was administered intravenously to soften the cervix. At 18:13, the uterine orifice was opened completely. At 18:15, the fetus was delivered. The newborn′s apgar score was 10–10, and the body weight was 2650 g. At 18:25, the placenta was delivered. Uterine contraction was normal, and the perineum was intact. The vital signs during delivery are shown in Table 1.

**Table 1:** Vital signs during delivery.

Point of time	Heart rate (bpm)	Blood pressure (mmHg)	Oxygen saturation (%)	Complaint
Enter the operating room	106	126/65	99	None
At the end of the first stage of labor	110	151/75	99	None
At the end of the second stage of labor	101	158/75	99	None
At the end of the third stage of labor	101	134/68	99	None
Leave the operating room	104	122/63	99	None
In transit	103	126/67	99	None
Enter ICU	95	125/59	100	None

**Figure 2:**
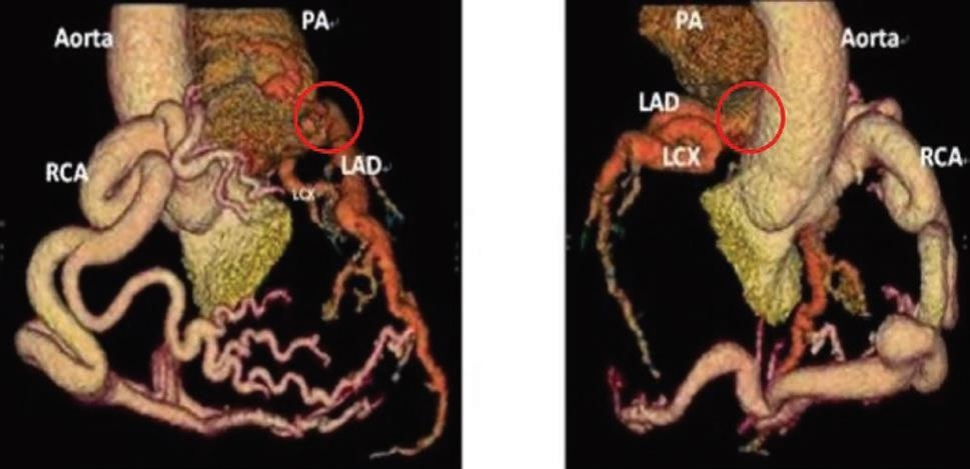
Coronary CTA: front view (left) and rear right view (right). RCA: right coronary artery; PA: pulmonary artery; LCX: left circumflex; LAD: left anterior descending artery. The red circle indicates the origin of the left coronary artery from the pulmonary artery.

**Figure 3:**
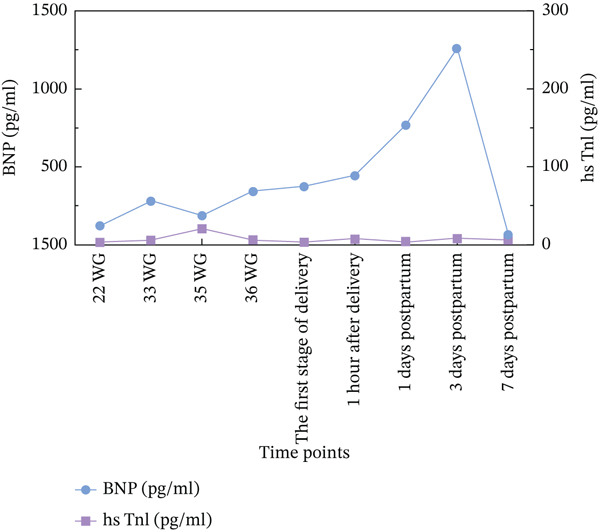
Levels of BNP and hsTnI during pregnancy and after delivery. BNP: B‐type natriuretic peptide. WG: weeks of gestation; hsTnI, high‐sensitivity cardiac Troponin I.

After delivery, the patient entered the ICU for 24‐h monitoring and received anti‐infection treatment. She returned to the general ward on the 1st day after delivery, and coronary CTA showed an anomalous left coronary artery originating from the left pulmonary artery (Figure 2). On the 4th day after delivery, the laboratory examination and echocardiography were rechecked, and diuretics were additionally used. Finally, the patient was discharged. Then outpatient re‐examination and testing were performed within 3 days after discharge. Echocardiography findings during pregnancy and after delivery are shown in Table 2. The levels of BNP and hsTnI during pregnancy and after delivery are shown in Figure 3.

**Table 2:** Echocardiography during pregnancy and after delivery.

Time point	EF(%)	LVEDD(mm)	LAD(mm)	VCW(mm)	Echocardiography conclusion
13^+1^	56	57	40	2.2	Mild mitral regurgitation
22^+1^	68	57	45∗58∗62	4.5	Moderate mitral regurgitation
33	60	59	44	4.8	Moderate mitral regurgitation
35^+6^	64	56	43∗49∗60	5.6	Moderate mitral regurgitation
3 days postpartum	64	57	49∗53∗60	8.2	Severe mitral regurgitation
7 days postpartum	53	56	38∗48∗58	2.1	Mild mitral regurgitation

Abbreviations: LAD, left atrial diameter; LVEDD, left ventricular end diastolic dimension; VCW, vena contracta width.

## 2. Discussion

ALCAPA can have a serious impact on cardiac function [4]. This is due to the establishment of collateral circulation between coronary arteries and the dilation of normally originating coronary arteries. These changes reduce coronary artery perfusion pressure, leading to varying degrees of myocardial ischemia. Even in asymptomatic patients, chronic myocardial ischemia persists. Over time, this can lead to ischemic cardiomyopathy and malignant arrhythmias originating from myocardial scar tissue, further worsening cardiac function. This is also the main cause of death in adults with abnormal origin of coronary arteries. The average age of survival is 35 years due to approximately 80%–90% of ALCAPA patients experiencing sudden death [5]. During pregnancy, a series of physiological changes will occur [6], mainly manifested as an increase in total blood volume, cardiac output, and heart rate, increasing myocardial oxygen consumption. Once the opening of the left coronary artery in pregnant patients originates from the pulmonary artery, the risk of myocardial ischemia will increase several times, and patients will be highly prone to serious symptoms of myocardial ischemia and heart failure. Therefore, such patients have a very high risk of pregnancy.

### 2.1. About the Timing of Terminating Pregnancy

If the symptoms of myocardial ischemia gradually become worse due to the increase in cardiac load and oxygen consumption in late pregnancy, timely termination of pregnancy, in addition to oxygen inhalation and bed rest, should be considered based on fetal conditions. The patient in this case report was pregnant for 37 weeks with preeclampsia, and the fetus was already full term. Therefore, it is recommended to terminate the pregnancy as soon as possible.

### 2.2. About the Method of Terminating Pregnancy

There has been no unified international recommendation on the method of terminating pregnancy for such patients. During natural delivery, factors such as prolonged uterine contraction (causing hemodynamic changes) and first‐stage labor pain (increasing myocardial oxygen consumption) can elevate the risk of myocardial ischemia, infarction, or even heart failure. Therefore, to reduce cardiac burden, cesarean delivery is often the preferred mode of delivery for most of these patients [7].

After many MDT discussions on pregnancy with heart disease in our hospital, we knew that the patient was a multiparous woman who had had natural delivery twice, and the labor process this time was estimated to be relatively short. Labor analgesia was feasible in the first stage of labor to reduce the patient′s pain and stimulation. Active midwifery care could be given in the second stage of labor to avoid abdominal pressure. The whole labor process was closely monitored in the operating room and escorted by the MDT (anesthesiologist, cardiologist, cardiac surgeon, neonatal pediatrician, radiologist, and obstetrician).

This patient had adult‐type ALCAPA, and the patient′s myocardial ischemia symptoms were relieved to a certain extent. At ordinary times, she has no symptoms when myocardial ischemia does not occur. Insufficient blood supply to the subendocardial myocardium will lead to the proliferation of elastic fiber tissue, causing extensive fibrosis, and even subendocardial calcification. At the same time, it will also cause dysfunction of the papillary muscle, fusion and shortening of the mitral chordae tendineae. Left ventricular fibrosis can also result in the enlargement of the cardiosphere and mitral annulus. As a result, mitral insufficiency of different degrees will occur. In this case report, echocardiography indicated mitral regurgitation in the patient, consistent with the aforementioned pathophysiological alterations. Concurrently, increased blood volume during pregnancy and the postpartum surge in cardiac blood volume elevated the cardiac load, leading to further dilation of the already enlarged mitral annulus. Consequently, the severity of mitral regurgitation worsened by the third day after delivery, progressing to severe mitral regurgitation. Following the peak in cardiac blood flow by the seventh day postpartum, cardiac function demonstrated significant improvement compared with the pre‐delivery state, with echocardiography revealing only mild mitral regurgitation.

### 2.3. Prenatal Screening and Postpartum Management

Regular prenatal examinations facilitate the timely detection of abnormalities in patients. Particularly for asymptomatic adult‐type ALCAPA, screening via electrocardiography and echocardiography enables the early identification of such high‐risk individuals, allowing for targeted risk stratification and multidisciplinary care planning. Postpartum, the valvular changes and cardiac function status indicated by echocardiography, combined with indicators such as BNP, contribute to clarifying the patient′s cardiac tolerance to hemodynamic fluctuations and overall cardiac performance, thereby aiding in the prevention of adverse cardiovascular events.

## 3. Conclusion

Under the collaboration of MDT, personalized treatment plans were selected for the patient, and the optimal timing and method of terminating pregnancy were determined, preventing the occurrence of serious cardiovascular adverse events and ensuring the mother–infant safety.

## Funding

Beijing Municipal Health Commission (2024‐2‐2068).

## Ethics Statement

This study was approved by the Ethics Committee of the Beijing Anzhen Hospital affiliated to Capital Medical University (2024087X). Patient consent for photograph and publication was obtained.

## Conflicts of Interest

The authors declare no conflicts of interest.

## Data Availability

The data supporting the findings of this study are available from the corresponding author upon reasonable request.

## References

[1] Vilá Mollinedo L. G. , Jaime Uribe A. , Aceves Chimal J. L. , Martínez-Rubio R. P. , and Hernández-Romero K. P. , Case Report: ALCAPA Syndrome: Successful Repair With an Anatomical and Physiological Alternative Surgical Technique, F1000Research. (2016) 5, 10.12688/f1000research.8823.1, 2-s2.0-85011049812. PMC4975369 27547381

[2] Peña E. , Nguyen E. T. , Merchant N. , and Dennie C. , ALCAPA Syndrome: Not Just a Pediatric Disease, Radiographics. (2009) 29, no. 2, 553–565, 10.1148/rg.292085059, 2-s2.0-66149151464, 19325065. 19325065

[3] Dodge-Khatami A. , Mavroudis C. , and Backer C. L. , Anomalous Origin of the Left Coronary Artery From the Pulmonary Artery: Collective Review of Surgical Therapy, Annals of Thoracic Surgery. (2002) 74, no. 3, 946–955, 10.1016/s0003-4975(02)03633-0, 2-s2.0-0036712672, 12238882. 12238882

[4] Yuan X. , Li B. , Sun H. , Yang Y. , Meng H. , Xu L. , Song Y. , and Xu J. , Surgical Outcome in Adolescents and Adults With Anomalous Left Coronary Artery From Pulmonary Artery, Annals of Thoracic Surgery. (2018) 106, no. 6, 1860–1867, 10.1016/j.athoracsur.2018.05.051, 2-s2.0-85054383353, 29928853. 29928853

[5] Alexi-Meskishvili V. , Berger F. , Weng Y. , Lange P. E. , and Hetzer R. , Anomalous Origin of the Left Coronary Artery From the Pulmonary Artery in Adults, Journal of Cardiac Surgery. (1995) 10, no. 4, 309–315, 7549188, 10.1111/j.1540-8191.1995.tb00617.x, 2-s2.0-0029092385. 7549188

[6] Chandra M. and Paray A. A. , Natural Physiological Changes During Pregnancy, Yale Journal of Biology and Medicine. (2024) 97, no. 1, 85–92, 10.59249/JTIV4138. 38559455 PMC10964813

[7] Wang H. , Liang Z. , Zhang G. , Fang H. , and Li D. , A Rare Case of an Adult Pregnant Patient With the Left Coronary Artery Originating From the Pulmonary Artery: Successful Management and Healthy Maternal-Fetal Outcome, Heart Surgery Forum. (2023) 26, no. 5, E441–E448, 10.59958/hsf.6407, 37920079. 37920079

